# Systemic Corticosteroid Administration in Coronavirus Disease 2019 Outcomes: An Umbrella Meta-Analysis Incorporating Both Mild and Pulmonary Fibrosis–Manifested Severe Disease

**DOI:** 10.3389/fphar.2021.670170

**Published:** 2021-05-26

**Authors:** Bin Cheng, Jinxiu Ma, Yani Yang, Tingting Shao, Binghao Zhao, Linxiang Zeng

**Affiliations:** ^1^ Department of Emergency and Critical Care Medicine, The Second Affiliated Hospital of Nanchang University, Nanchang, China; ^2^ Department of Respiratory and Critical Care Medicine, The Second Affiliated Hospital of Nanchang University, Nanchang, China; ^3^ Department of Neurosurgery, Peking Union Medical College Hospital, Chinese Academy of Medical Sciences and Peking Union Medical College, Beijing, China

**Keywords:** corticosteroids, coronavirus, COVID-19, critically ill/pulmonary fibrosis manifested, umbrella meta-analysis

## Abstract

**Background:** Effective treatments for coronavirus disease 2019 (COVID-19) are urgently needed. The real role of corticosteroid use in COVID-19 has long been of interest and is disputable.

**Methods:** We aimed to quantitatively reevaluate the efficacy of corticosteroids on COVID-19. Databases were searched for eligible meta-analyses/systematic reviews with available outcome data. For each association, we estimated the summary effect size with fixed- and random-effects models, 95% confidence intervals, and 95% prediction intervals. Heterogeneity, Egger’s test, evidence of small-study effects and excess significance bias, and subgroup analyses were rigorously evaluated.

**Results:** Intended outcomes of 12 eligible studies were mortality, clinical improvement, hospitalization, mechanical ventilation (MV), adverse events (AEs), intensive care unit (ICU) stay, hospital stay, virus clearance time (VCT), and negative conversion. Corticosteroid administration was associated with a 27% risk reduction in MV [hazard ratio (HR): 0.73 (0.64–0.83)] and a 20% reduction in mortality of critically ill/severe COVID-19 patients [HR: 0.80 (0.65–0.98)]. Interestingly, shorter ICU stays and, conversely, potentially longer hospital stays, a longer VCT, and a longer time to negative conversion were associated with corticosteroid use. There was no significant impact of different corticosteroid doses on mortality. Only one study showed slightly excess significant bias. Caution should be applied given the weak nature of the evidence, and it has been confirmed by sensitivity analyses too.

**Conclusion:** This umbrella study found benefits from corticosteroids on MV and especially the mortality of critically ill/severe patients with shorter ICU stays but prolonged hospital stays and VCT. The benefits and harms should be reevaluated and balanced before corticosteroids are cautiously prescribed in clinical practice.

## Introduction

At the end of 2019, a novel coronavirus, severe acute respiratory syndrome coronavirus (SARS-CoV-2), broke out in Wuhan, China (Zhou et al., 2020); by March 11, 2020, the outbreak was declared a pandemic of coronavirus disease 2019 (COVID-19) by the World Health Organization (WHO) (Zhang et al., 2020). To date, COVID-19 has affected millions worldwide, while no pronouncedly effective treatment has been established (Holshue et al., 2020).

The immune response is acknowledged to be a key factor in COVID-19, of which the first phase is characterized by fever, cough, and high viral loads; the next stage is marked by lung inflammation and respiratory failure accompanied by a decreased viral load; and the last stage shows an uncontrolled hyperinflammatory response, subsequent pulmonary fibrosis, and multiorgan dysfunction with a high mortality risk (Tay et al., 2020). As the main immunomodulatory agent used for clinical management, corticosteroids are thought to be a candidate for COVID-19 treatment (Ho et al., 2003; Tay et al., 2020). Both benefits and poor outcomes have been reported in the previous literature (Budhathoki et al., 2020; Cano et al., 2020; Cheng et al., 2020; Lu et al., 2020; Pasin et al., 2020; Pei et al., 2020; Sarkar et al., 2020; Siemieniuk et al., 2020; Sterne et al., 2020; Tlayjeh et al., 2020; van Paassen et al., 2020; Ye et al., 2020), and rigorous data on its efficacy are still limited, which potentially will stimulate clinical research and address this controversy. The United Kingdom–based Randomized Evaluation on COVID-19 Therapy (RECOVERY) trial reported reliable findings from 6,425 patients who were randomized to receive 6 mg/d dexamethasone or standard of care (SOC). Dexamethasone resulted in a mortality reduction of 2.8% compared with SOC [22.9 vs. 25.7%, relative risk (RR): 0.83, 95% confidence interval (95% CI): 0.75–0.93], and more benefits could be found for the mortality of patients receiving invasive mechanical ventilation [29.3 vs. 41.4%, respectively, RR: 0.64, 95% CI: 0.51–0.81] (Horby et al., 2020). The results of the meta-analysis were also controversial since a previous meta-analysis demonstrated that COVID-19 patients who used corticosteroids were more likely to develop critical illness and had a higher mortality and longer hospital stays (Wang et al., 2020). Another meta-analysis showed a significant reduction in short-term mortality [odds ratio (OR): 0.72, 95% CI: 0.57–0.87] and the need to receive mechanical ventilation (MV) compared to SOC, although the data were sparse to draw firm conclusions (van Paassen et al., 2020). To our knowledge, there has been little attempt to summarize the data from existing secondary studies.

Considering the great impact of the pandemic and the inconsistency in these findings, this study aimed to comprehensively summarize the emerging secondary evidence (meta-analyses with/without systematic reviews) being reported worldwide. We sought to (i) determine the association between outcomes of interest and corticosteroid use; (ii) better understand the strength of the data and the extent of potential bias in the claimed results via the umbrella method; (iii) show reliable evidence by summarizing abundant sample sizes, totally analyzing the heterogeneity and performing subgroup analyses; and (iv) provide a potential effective treatment for COVID-19.

## Methods

We rigorously conducted this umbrella meta-analysis following prior guidelines (Aromataris et al., 2015) (Supplementary Appendix File A1). The protocol has been registered on the INPLASY website (https://inplasy.com/register/) (ID: INPLASY202110116, doi: *10.37766/inplasy 2021.1.0116*) (Supplementary Appendix File A2).

### Search Strategy

We searched PubMed, EMBASE, Cochrane Library, and preprint platforms for related systematic reviews and/or meta-analyses from database inception to December 1, 2020, with no language restrictions. The search keywords used were as follows: COVID-19, corticosteroids, systematic review, and meta-analysis. Detailed search information can be found in Supplementary Appendix File A3. A manual search of reference lists from the retrieved studies was also performed.

### Selection Criteria

Articles were initially reviewed through titles and abstracts. Then, full texts of potentially eligible studies were examined by two authors, and any discrepancies were resolved with a discussion involving a third author (Binghao Zhao) who is familiar with COVID-19 epidemiology and evidence-based medicine.

The eligible criteria were as follows:

**Population**: Hospitalized patients with confirmed COVID-19.

**Intervention**: Reasonable corticosteroid use (methylprednisolone, dexamethasone, prednisone, corticoids, and steroids) during the hospital stay.

**Comparison**: No corticosteroid use, including SOC and placebo.

**Outcomes**: Mortality, MV, hospital stay, virus clearance time (VCT), intensive care unit (ICU) stay, adverse events (AEs), clinical improvement, hospitalization, and negative conversion. Eligible studies should report at least one of the intended outcomes.

**Study design**: Meta-analysis with/without a systematic review with available data. Associations with intended outcomes of eligible secondary studies should include at least two primary studies, except network meta-analyses.

Only studies with the most complete information could be included. We excluded single-arm meta-analyses; meta-analyses involving pregnant or pediatric patients with different presentations of COVID-19 in these populations; and meta-analyses involving organ transplant recipients or inflammatory or rheumatologic patients using long-term corticosteroids.

### Data Extraction

Data extraction was performed independently by two authors, and any disagreements were resolved by discussion with a third author. For each eligible article, we recorded the first author, publication year, number of included studies, number of participants, comparisons, study design, quality assessment methods, subgroup data, searching and registration information, pooled risk estimates [RR, OR, hazard ratio (HR), incident RR, mean difference (MD), and weighted MD (WMD)], and 95% CI. For each primary study from the eligible meta-analyses, the first author, number of cases and subjects, maximally adjusted risk estimate, and 95% CI were extracted for further analysis if available.

### Assessment of Small-Study Effects

Egger’s regression asymmetry test was conducted to help identify small-study effects (Sterne et al., 2011). Although small-study effects can indicate publication bias and other reporting biases, they can also reflect genuine chance, heterogeneity, or other causes for the differences between small and large studies. We calculated the standard error (SE) of the study with the largest sample size of each meta-analysis to determine whether larger estimates of effect size were predicted by small studies compared to large studies. If the *p* value for Egger’s test was <0.10 and the largest study had a smaller effect size than the summary effect size, criteria for the existence of small-study effects were fulfilled (Sterne et al., 2011).

### Evidence of Excess Significance Bias

An excess significance test was conducted to investigate whether the observed number of studies (O) with nominally significant results (*p* < 0.05, “positive” results) was larger than the expected number of significant results (E) (Ioannidis and Trikalinos, 2007). In each meta-analysis, E is calculated from the sum of the statistical power estimates for each component study. We used the effect size of the largest study in a meta-analysis and the pooled results based on fixed- and random-effects models as plausible effect sizes to estimate the power of each component study (Ioannidis, 2013). The estimate from the largest study should be closer to the true estimate than the results of less precise studies with small-study effects. The statistical power was computed by using a noncentral t distribution (Lubin and Gail, 1990). The excess significance test was regarded as positive if the *p* value was <0.10, given that O > E.

### Reviewing the Current Evidence

Statistically significant associations between corticosteroid use and outcomes of interest were categorized into four levels: strong, highly suggestive, suggestive, and weak using specific criteria. For strong evidence, *p* < 10^–6^, number of cases >1,000, I^2^ < 50%, *p* < 0.05 of the largest study in the meta-analysis, and 95% prediction interval (PI) excludes the null value, small-study effects (*p* > 0.1 for Egger’s test), and excess significance bias (*p* > 0.1). For highly suggestive evidence, *p* < 10^–6^, number of cases >1,000, and *p* < 0.05 of the largest study in the meta-analysis. For suggestive evidence, *p* < 10^–3^ and number of cases >1,000. For weak evidence, the sole criterion was *p* < 0.05 (Kalliala et al., 2017). When *p* > 0.05, there was no association. For the number of cases with unknown outcomes, the quality was judged according to the total population; for both cases and total populations with unknown outcomes, the quality was weak regardless of whether *p* < 0.05.

### Statistical Analysis

For each meta-analysis, we also estimated the summary effect size and its 95% CI using both fixed-effects and random-effects models (Kalliala et al., 2017). After addressing the uncertainty of the estimated summary effect in the random-effects model and the heterogeneity between the studies, the 95% CI was calculated to predict the expected effect size range in the new original studies (Riley et al., 2011). For the largest dataset of each meta-analysis, we calculated the SE of the effect size and determined whether the SE was less than 0.10. For binary variables, HRs and 95% CIs were used to pool the results from each meta-analysis together based on the extracted RR, OR, HR, and incident RR because the influence of time on mortality and MV could be properly considered, and there were still heterogeneity and missing information about sample size across the involved populations of each study. For continuous variables, MDs and WMDs were used. We used the I^2^ statistic and derived *p* values of the Cochran’s Q statistic to assess heterogeneity among studies (Riley et al., 2011). When I^2^ was greater than 50 or 75%, the heterogeneity was considered to be substantial or considerable, respectively. We also calculated the 95% CI of I^2^ to assess the uncertainty around the heterogeneity estimates (Ioannidis et al., 2007). Sensitivity analyses were performed to detect the source of heterogeneity and other bias if necessary, hence, to check the robustness of these findings.

Data analyses were performed using Stata (version 14.0) and R software (version 3.5.3) with the “forest” public packages. A two-sided *p* value <0.05 was considered statistically significant except for in the special cases described above.

## Results

### Literature Search

**FIGURE 1:**
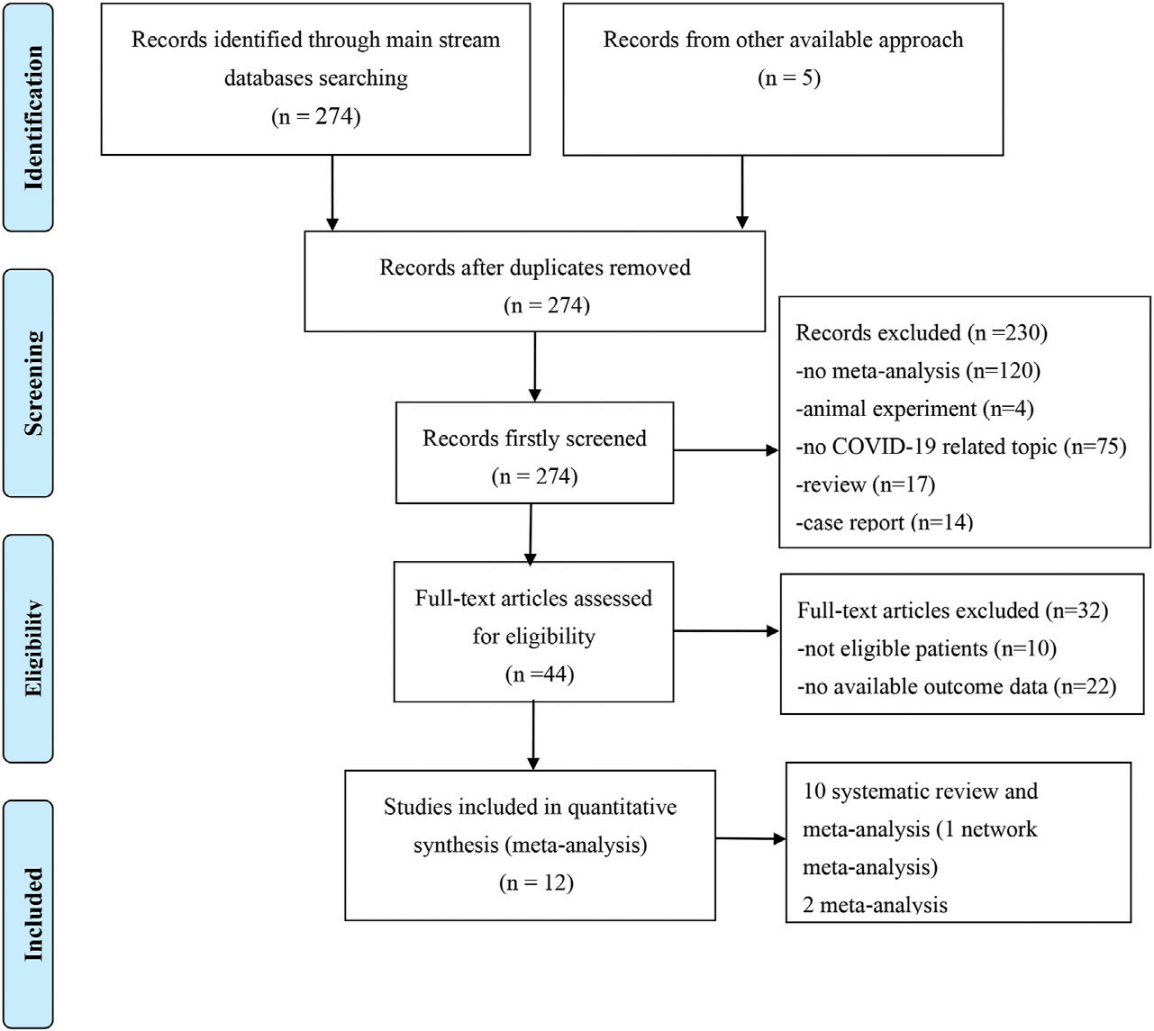
Flow chart of the study selection process.

We obtained 279 records from the electronic database search (274 from PubMed, EMBASE, and Cochrane Library; 5 from the manual search of the reference list). After excluding the five duplicated studies, 230 studies were removed by screening titles and abstracts and 32 were removed after assessment of the full text. Ultimately, 12 meta-analyses with/without systematic reviews were included for further analysis (Figure 1). No studies from preprint platforms were included, considering the lack of a strict peer-review process.

### Characteristics of the Included Studies

The main characteristics of the included studies are summarized in Table 1. Among 12 eligible studies, all of them (Budhathoki et al., 2020; Cano et al., 2020; Cheng et al., 2020; Lu et al., 2020; Pasin et al., 2020; Pei et al., 2020; Sarkar et al., 2020; Siemieniuk et al., 2020; Sterne et al., 2020; Tlayjeh et al., 2020; van Paassen et al., 2020; Ye et al., 2020) reported mortality; six studies (Budhathoki et al., 2020; Cheng et al., 2020; Pasin et al., 2020; Siemieniuk et al., 2020; Tlayjeh et al., 2020; van Paassen et al., 2020) reported MV; five studies (Budhathoki et al., 2020; Cheng et al., 2020; Lu et al., 2020; Sarkar et al., 2020; Siemieniuk et al., 2020) reported the outcome duration of hospital stay; two studies (Cheng et al., 2020; Sarkar et al., 2020) reported VCT; two studies (Budhathoki et al., 2020; Cheng et al., 2020) reported clinical improvement; one study (Cheng et al., 2020) reported ICU stay duration; one study (Sterne et al., 2020) reported AE outcomes; one study (Budhathoki et al., 2020) reported negative conversion time; and one study (Budhathoki et al., 2020) reported hospitalization. Notably, Budhathoki et al. (Budhathoki et al., 2020) conducted subgroup analyses on invasive MV and non-invasive MV. Cano et al. (Cano et al., 2020), Cheng et al. (Cheng et al., 2020), Pasin et al. (Pasin et al., 2020), Sterne et al. (Sterne et al., 2020), Tlayjeh et al. (Tlayjeh et al., 2020), and Ye et al. (Ye et al., 2020) analyzed mortality for severe vs. non-severe COVID-19, for high dose vs. low dose of corticosteroids, for MV requirement vs. no MV requirement, and for critically ill COVID-19. Cheng et al. (Cheng et al., 2020) reported VCT for severe and non-severe COVID-19. The drug dose was based on defined cutoffs: 15 mg/d dexamethasone, 400 mg/d hydrocortisone, and 1 mg/kg methylprednisolone or equivalent corticosteroids, as described in the primary studies (Annane et al., 2017). Patients who needed MV were assigned to critically ill/severe COVID-19, and patients with no MV or no oxygen requirement were assigned to non-severe COVID-19. The study number of each comparison varied from 1 to 27, and Sarkar et al. (Sarkar et al., 2020) conducted the largest review incorporating the most patients. Among the 12 articles, 10 (Budhathoki et al., 2020; Cano et al., 2020; Cheng et al., 2020; Lu et al., 2020; Pei et al., 2020; Sarkar et al., 2020; Siemieniuk et al., 2020; Tlayjeh et al., 2020; van Paassen et al., 2020; Ye et al., 2020) were systematic reviews and meta-analyses, 2 (Pasin et al., 2020; Sterne et al., 2020) were meta-analyses, and 1 (Siemieniuk et al., 2020) was a network meta-analysis; three studies (Pasin et al., 2020; Siemieniuk et al., 2020; Sterne et al., 2020) involved only randomized controlled trials (RCTs), while the others included not only RCTs but also observational studies or case series. All studies applied accurate quality assessment tools to evaluate the studies’ quality; however, only two studies (Pasin et al., 2020; Sterne et al., 2020) provided clear registration information. All eligible meta-analyses used summary-level data based on the published literature, and none provided or used individual participant data. The publication year was 2020.

**TABLE 1:** Main characteristics of included meta-analyses and/or systematic reviews that evaluate systematic corticosteroid use on COVID-19.

Publication	Outcomes	No. of studies	No. of events	No. of populations	Comparison	Included studies	Results (95% CI)	Quality assessment	Registration	Search deadline
Budhathoki et al. (2020) (Nepal)	Mortality	14	461/195	2083/2368	Steroids + SOC vs. SOC or placebo	Case series, observational studies, RCTs	RR: 2.01 (1.12–3.63); RD: 0.10 (0.02–0.17)	NHLBI tool	No registration	June 3, 2020
	Discharge rate	9	280/645	505/885			RR: 0.79 (0.63–0.99); RD: -0.13 (-0.26–-0.01)			
	Hospitalization	4	35/55	96/137			RR: 1.28 (0.27–6.17)			
	Clinical improvement	9	552/1576	801/1754			OR: 0.24 (0.13–0.43)			
	MV	6	133/90	346/338			OR: 1.44 (0.35–5.92)			
	Hospital stay	4	NA	885/1871			MD: 4.19 (2.57–5.81)			
	Negative conversion	4	NA	272/634			MD: 2.42 (1.31–3.53)			
Cano et al. (2020) (America)	Mortality	27	1193/1494	4919/8735	Corticosteroids vs. SOC or placebo	Respective studies	OR: 2.30 (1.45–3.63)	ROBINS-I tool and Cochrane Handbook	No registration	July 20, 2020
Cheng et al. (2020) (China)	Clinical improvement	1	98/46	98/46	Corticosteroids vs. SOC or placebo	Observational studies and case series	RR: 1.30 (0.98–1.72); WMD: 1.69 (-0.24–3.62)	NOS and GRADE approach	CRD42020184545^†^	July 30, 2020
	Mortality	6	235/127	985/1364			RR: 1.59 (0.69–3.66)			
	VCT	5	NA	92/155			WMD: 1.01 (-0.91–2.92)			
	MV	1	26/20	26/20			RR: 0.35 (0.10–1.18)			
	Hospital stay	3	MA	169/121			WMD: -3.17 (-7.37–1.04)			
	ICU stay	1	NA	26/20			WMD: -6.50 (-7.63–-5.37)			
Lu et al. (2020) (China)	Mortality	4	94/58	329/408	Corticosteroids vs. SOC or placebo	Observational studies and RCTs	RR: 2.00 (0.69–5.75)	NOS, Cochrane Handbook, and GRADE approach	No registration	March 31, 2020
	Duration of fever	1	26/20	26/20			WMD: -3.23 (-3.56–-2.90)			
	Lung inflammation absorption	1	51/21	51/21			WMD: -1.00 (-2.91–0.91)			
	Hospital stay	2	NA	153/212			WMD: 2.43 (1.42–3.43)			
Pasin et al. (2020) (multiple nations)	Mortality	5	727/1336	2835/4857	Corticosteroids vs. SOC or placebo	RCTs	RR: 0.89 (0.82–0.96)	Cochrane Handbook	CRD42020197509	Not reported
	MV	3	126/311	2329/4544			RR: 0.75 (0.60–0.94)			
Pei et al. (2020) (China)^‡^	Mortality	5	252/266	352/591	Corticosteroids vs. no corticosteroids	Observational studies and RCTs	OR: 2.43 (1.44–4.10)	NOS and Jadad score	No registration	April 7, 2020
Sarkar et al. (2020) (India)	Mortality	12	1306/1711	5611/10143	Corticosteroids vs. SOC or placebo	Observational studies and RCTs	OR: 1.94 (1.11–3.40)	ROBINS-I tool, ROB 2.0 tool, and GRADE approach	No registration	August 19, 2020
	Hospital stay	6	—	1134/1598			MD: 1.18 (-1.28–3.64)			
	VCT	2	—	82/69			MD: 1.42 (-0.52–3.37)			
Siemieniuk et al. (2020) (multiple nations)^§^	Mortality	1	—	—	Corticosteroids vs. SOC or placebo	RCTs	OR: 0.89 (0.64–1.40)	Cochrane Handbook and GRADE approach	No registration	August 10, 2020
	MV	1					OR: 0.73 (0.58–0.92)			
	Hospital stay	1					MD: -0.99 (-1.36–-0.64)			
Sterne et al. (2020) (multiple nations)	Mortality	7	222/425	678/1025	Corticosteroids vs. SOC or placebo	RCTs	OR: 0.70 (0.48–1.01)	Cochrane Handbook	CRD42020197242	April 6, 2020
	AEs	6	64/80	354/342			OR: 0.84 (0.54–1.20)			
Tlayjeh et al. (2020) (Arabia)**^||^**	Mortality	10	Not reported	Total 13564	Corticosteroids vs. SOC or placebo	Observational studies and RCTs	HR: 0.91 (0.71–1.16)	Cochrane Handbook, ROBINS-I tool, and GRADE approach	No registration	July 20, 2020
	MV	3	Not reported	Total 5768			ES: 0.74 (0.50–1.09)			
van Paassen et al. (2020) (Netherlands)	Mortality	22	Not reported	Total 14187	Corticosteroids vs. SOC or placebo	Observational studies and RCTs	OR: 0.72 (0.57–0.87)	NOS and Cochrane Handbook	No registration	October 1, 2020
	MV	7	Not reported	Total 939			RR: 0.70 (0.54–0.91)			
Ye et al. (2020) (multiple nations)	Mortality	2	Not reported	227/104	Corticosteroids vs. SOC or placebo	Observational studies and RCTs	HR: 2.30 (1.00–5.29)	NOS and Cochrane Handbook	No registration	April 25, 2020

**Abbreviations:** AEs, adverse events; 95% CI, 95% confidence interval; COVID-19, coronavirus disease 2019; ES, estimate size/effect size; HR, hazard ratio; ICU, intensive care unit; MD, mean difference; MV, mechanical ventilation; NA, not available; NHLBI tool, National Heart, Lung, and Blood Institute tool; NOS, The Newcastle–Ottawa Scale; OR, odds ratio; RCT, randomized controlled trial; ROBINS-I, Risk Of Bias In Non-randomized Studies of Interventions; RR, relative risk; SOC, standard of care; VCT, virus clearance time; WMD, weighted mean difference.

^†^ Registration number headed by CRD was registered on the PROSPERO website.

^‡^ Results in the study of Pei et al. were calculated according to the data provided in that study.

^§^ The study of Siemieniuk et al. is a network meta-analysis, where clear number of patients in each group was not reported.

^||^ Detailed number of patients was not available in this study.

### Summary of the Main Results of the Outcomes

The 12 meta-analyses with 12 kinds of outcomes and 34 comparisons were summarized and reperformed using both fixed- and random-effects models (Table 2). Both fixed and random *p* values <0.05 were found in 13 comparisons that incorporated outcomes of mortality, discharge rate, clinical improvement, hospital stay, negative conversion, and MV. Four comparisons (discharge rate, mortality, MV) under the random-effects model illustrated significant benefits from corticosteroids, and nine comparisons (mortality, clinical improvement, hospital stay, negative conversion) with the random-effects model illustrated harms. The largest study with the smallest SE for each comparison suggested that 25 of 34 comparisons were significant at a *p* value <0.05. After excluding null values of 95% CI, no association between corticosteroid use and outcomes of interest was found. Ten comparisons (37%, 10/27) of outcomes revealed low heterogeneity (I^2^ ≤ 50%), 5 (18.5%, 5/27) revealed substantial heterogeneity (50% < I^2^ ≤ 75%), and 12 (44.4%, 12/27) showed considerable heterogeneity estimates (I^2^ > 75%) (Table 3).

**TABLE 2:** Descriptions of the risk estimates of 34 primary comparisons included in umbrella meta-analysis.

Publication	Outcomes	No. of studies	Fixed result	Random result	Largest study result*	Fixed *p* value	Random *p* value	95% PI	Evidence grade
Budhathoki et al. (2020) (Nepal)	Mortality	14	RR: 2.34 (2.03–2.70)	RR: 2.01 (1.12–3.63)	RR: 5.95 (4.26–8.31)	<0.01	0.02	0.19–20.86	Weak
	Discharge rate	9	RR: 0.81 (0.74–0.88)	RR: 0.79 (0.63–0.99)	RR: 0.84 (0.76–0.92)	<0.01	0.04	0.43–1.46	Weak
	Hospitalization	4	RR: 1.48 (0.91–2.40)	RR: 1.29 (0.27–6.16)	RR: 9.68 (0.60–155.02)	0.11	0.75	0–997.35	Not confirmed
	Clinical improvement	9	OR: 0.81 (0.77–0.85)	OR: 0.81 (0.71–0.91)	OR: 0.50 (0.36–0.71)	<0.01	< 0.01	0.56–1.15	Suggestive
	MV	6	OR: 1.43 (1.10–1.87)	OR: 1.36 (0.56–3.31)	OR: 23.77 (8.31–68.03)	<0.01	0.5	0.07–27.98	Not confirmed
	Hospital stay	4	WMD: 3.98 (3.20–4.77)	WMD: 4.19 (2.57–5.81)	MD: 3.98 (2.92–5.04)	<0.01	< 0.01	-13.65	Suggestive
	Negative conversion	4	WMD: 2.42 (1.31–3.53)	WMD: 2.45 (1.11–3.79)	MD: 2.50 (1.14–3.86)	<0.01	< 0.01	-7.65	Weak
Cano et al. (2020) (America)	Mortality	27	OR: 1.21 (1.13–1.30)	OR: 1.79 (1.30–2.46)	OR: 0.86 (0.76–0.97)	<0.01	< 0.01	0.44–7.35	Highly suggestive
Cheng et al. (2020) (China)	Clinical improvement	1	RR: 1.30 (0.92–1.72)	RR: 1.30 (0.92–1.72)	RR: 1.30 (0.92–1.72)	0.05	0.05	—	Not confirmed
	Mortality	6	RR: 2.26 (1.89–2.70)	RR: 1.30 (0.49–3.44)	RR: 5.95 (4.26–8.31)	<0.01	0.6	0.04–41.64	Not confirmed
	VCT	5	WMD: 2.10 (1.39–2.80)	WMD: 1.91 (-0.88–4.70)	WMD: 0.50 (-3.55–4.55)	<0.01	0.18	-20.6	Not confirmed
	MV	1	RR: 0.35 (0.10–1.18)	RR: 0.35 (0.10–1.18)	RR: 0.35 (0.10–1.18)	—	—	—	Not confirmed
	Hospital stay	3	WMD: -4.08 (-4.82--3.35)	WMD: -2.69 (-9.82--3.92)	WMD: -3.00 (-3.34--2.66)	<0.01	0.43	-170.23	Not confirmed
	ICU stay	1	WMD: -6.50 (-7.63--5.37)	WMD: -6.50 (-7.63--5.37)	WMD: -6.50 (-7.63--5.37)	—	—	—	Weak
Lu et al. (2020) (China)	Mortality	4	RR: 2.03 (1.55–2.67)	RR: 2.00 (0.69–5.75)	RR: 6.03 (2.91–12.52)	<0.01	0.2	0.02–229.17	Not confirmed
	Duration of fever	1	WMD: -3.23 (-3.56–-2.90)	WMD: -3.23 (-3.56–-2.90)	WMD: -3.23 (-3.56–-2.90)	—	—	—	Weak
	Lung inflammation absorption	1	WMD: -1.00 (-2.91–-0.91)	WMD: -1.00 (-2.91–-0.91)	WMD: -1.00 (-2.91–-0.91)	—	—	—	Not confirmed
	Hospital stay	2	WMD: 2.43 (1.42–3.43)	WMD: 2.43 (1.42–3.43)	WMD: 2.00 (0.67–3.33)	<0.01	< 0.01	—	Weak
Pasin et al. (2020) (multiple nations)	Mortality	5	RR: 0.89 (0.82–0.96)	RR: 0.89 (0.82–0.96)	RR: 0.89 (0.81–0.98)	<0.01	< 0.01	0.78–1.01	Weak
	MV	3	RR: 0.95 (0.85–1.05)	RR: 0.94 (0.85–1.04)	RR: 0.72 (0.57–0.91)	0.29	0.24	0.48–1.83	Not confirmed
Pei et al. (2020) (China)	Mortality	5	OR: 2.63 (1.93–3.59)	OR: 2.43 (1.44–4.10)	OR: 2.58 (1.33–4.98)	<0.01	< 0.01	0.44–13.37	Weak
Sarkar et al. (2020) (India)	Mortality	12	OR: 1.37 (1.29–1.47)	OR: 1.72 (1.09–2.72)	OR: 0.86 (0.76–0.97)	<0.01	0.02	0.32–9.28	Weak
	Hospital stay	6	WMD: 2.01 (1.43–2.58)	WMD: 1.20 (-1.25–3.66)	MD: 4.00 (2.94–5.06)	<0.01	0.34	-16.91	Not confirmed
	VCT	2	WMD: 1.42 (-0.52–3.37)	WMD: 1.42 (-0.52–3.37)	MD: 2.40 (-1.13–5.93)	0.15	0.15	—	Not confirmed
Siemieniuk et al. (2020) (multiple nations)	Mortality	1	OR: 0.89 (0.64–1.40)	OR: 0.89 (0.64–1.40)	OR: 0.89 (0.64–1.40)	—	—	—	Not confirmed
	MV	1	OR: 0.73 (0.58–0.92)	OR: 0.73 (0.58–0.92)	OR: 0.73 (0.58–0.92)	—	—	—	Weak
	Hospital stay	1	MD: -0.99 (-1.36–-0.64)	MD: -0.99 (-1.36–-0.64)	MD: -0.99 (-1.36–-0.64)	—	—	—	Weak
Sterne et al. (2020) (multiple nations)	Mortality	7	OR: 0.78 (0.68–0.89)	OR: 0.81 (0.67–0.98)	OR: 0.64 (0.50–0.82)	<0.01	0.03	0.53–1.24	Weak
	AEs	6	OR: 0.82 (0.65–1.03)	OR: 0.78 (0.47–1.30)	OR: 0.44 (0.17–1.11)	0.08	0.34	0.18–3.34	Not confirmed
Tlayjeh et al. (2020) (Arabia)^†^	Mortality	10	HR: 0.88 (0.81–0.95)	HR: 0.91 (0.71–1.16)	HR: 0.70 (0.56–0.86)	<0.01	0.44	0.40–2.04	Not confirmed
	MV	3	HR: 0.81 (0.69–0.96)	HR: 0.73 (0.50–1.09)	ES: 0.77 (0.62–0.95)	0.01	0.12	0.01–60.99	Not confirmed
van Paassen et al. (2020) (Netherlands)^†^	Mortality	22	HR: 0.87 (0.80–0.95)	HR: 0.92 (0.75–1.13)	OR: 0.83 (0.74–0.92)	<0.01	0.43	0.44–1.94	Not confirmed
	MV	7	HR: 0.70 (0.55–0.89)	HR: 0.70 (0.54–0.91)	RR: 0.61 (0.38–0.98)	<0.01	<0.01	0.44–1.11	Weak
Ye et al. (2020) (multiple nations)	Mortality	2	HR: 2.30 (1.00–5.29)	HR: 2.30 (1.00–5.29)	HR: 2.12 (0.78–5.76)	<0.01	<0.01	—	Weak

**Abbreviations:** AEs, adverse events; 95% CI, 95% confidence interval; ES, estimate size/effect size; HR, hazard ratio; ICU, intensive care unit; MD, mean difference; MV, mechanical ventilation; NA, not available; OR, odds ratio; 95% PI, 95% prediction interval; RR, relative risk; VCT, virus clearance time; WMD, weighted mean difference.

* Estimate size/effect size and 95% confidence interval of the largest study (most with the smallest SE) in each included meta-analysis.

^†^ Given some number of patients in distinct groups were unknown in the study of Tlayjeh et al. and van et al., the pooled results were calculated through the hazard ratio.

**TABLE 3:** Evaluation of bias and heterogeneity of 34 primary comparisons evaluating systematic corticosteroid use on COVID-19.

Publication	Outcomes	No. of studies	I^2^ (95% CI)*	*p* heterogeneity*	Egger’s *p* value^†^	SD largest	SD fixed	SD random	Observed^‡^	E largest^‡^	E fixed^‡^	E random^‡^	*p* value^§^
Budhathoki et al. (2020) (Nepal)	Mortality	14	88.8% (89.1–94.8%)	*p* < 0.001	0.815	43.38	11.4	42.72	9	13.99	14	6.426	—
	Discharge rate	9	62.0% (21.0–81.0%)	*p* = 0.007	0.771	1.136	1.332	3.424	4	8.751	8.991	6.5	—
	Hospitalization	4	80.0% (48.0–93.0%)	*p* = 0.002	0.772	329.58	5.802	22.935	1	0.304	1.357	0.29	—
	Clinical improvement	9	76.0% (55.0–88.0%)	*p* < 0.001	0.317	11.443	1.032	1.032	8	3.681	9	9	—
	MV	6	88.0% (77.0–93.0%)	*p* < 0.001	0.3	200.381	5.137	18.347	3	4.572	4.128	0.756	—
	Hospital stay	4	70.1% (14.0–89.6%)	*p* = 0.018	0.661	21.043	20.911	43.154	4	4	4	3.996	—
	Negative conversion	4	14.4% (0–86.9%)	*p* = 0.320	0.4781	19.317	17.046	20.578	2	3.876	3.976	3.872	—
Cano et al. (2020) (America)	Mortality	27	89.0% (86.0–92.0%)	*p* < 0.001	0.03897	4.294	5.024	28.17	12	22.0914	21.978	20.614	—
Cheng et al. (2020) (China)	Clinical improvement	1	—	—	—	—	—	—	—	—	—	—	—
	Mortality	6	96.0% (93.0%-0.97)	*p* < 0.001	0.5223	43.38	10.015	36.474	4	5.994	5.994	0.63	—
	VCT	5	91.4% (82.9–95.7%)	*p* < 0.001	0.9977	17.533	5.653	22.372	2	0.395	4.995	1.84	—
	MV	1	—	—		—	—	—		—	—	—	
	Hospital stay	3	98.7% (97.7–99.2%)	*p* < 0.001	0.369	1.673	6.386	25.631	3	3	3	1.617	—
	ICU stay	1	—	—		—	—	—		—	—	—	
Lu et al. (2020) (China)	Mortality	4	91.0% (80.0–96.0%)	*p* < 0.001	0.6228	50.001	7.756	35.043	2	2.557	3.88	0.748	—
	Duration of fever	1	—	—	—	—	—	—	—	—	—	—	—
	Lung inflammation absorption	1	—	—	—	—	—	—	—	—	—	—	—
	Hospital stay	2	0	*p* = 0.603	—	10.201	9.796	9.796	2	1.8	1.998	1.998	0.112
Pasin et al. (2020) (multiple nations)	Mortality	5	0 (0–69.6%)	*p* = 0.603	0.3308	3.476	4.475	4.475	1	3.99	3.389	3.389	—
	MV	3	0 (0–86.3%)	*p* = 0.469	0.6173	6.384	4.41	3.643	0	2.802	2.826	0.995	—
Pei et al. (2020) (China)	Mortality	5	61.9% (0–85.7%)	*p* = 0.033	0.1141	15.413	13.004	20.838	4	2.518	4.914	3.301	—
Sarkar et al. (2020) (India)	Mortality	12	96.3% (94.8–97.4%)	*p* < 0.001	0.3671	4.294	5.763	52.191	9	9.818	12	6.226	—
	Hospital stay	6	92.5% (86.5–95.9%)	*p* < 0.001	0.515	21.043	15.334	32.134	4	6	5.994	3.761	—
	VCT	2	0	*p* = 0.517	—	19.729	12.194	12.194	0	0.726	0.8	0.8	—
Siemieniuk et al. (2020) (multiple nations)	Mortality	1	—	—	—	—	—	—	—	—	—	—	—
	MV	1	—	—	—	—	—	—	—	—	—	—	—
	Hospital stay	1	—	—	—	—	—	—	—	—	—	—	—
Sterne et al. (2020) (multiple nations)	Mortality	7	30.9% (0–70.5%)	*p* = 0.192	0.3292	2.59	2.211	3.263	1	6.972	6.937	5.363	—
	AEs	6	17.4% (39.3–88.4%)	*p* = 0.691	0.4461	3.837	2.557	5.586	0	4.416	3.401	1.584	—
Tlayjeh et al. (2020) (Arabia)	Mortality	10	82.2% (70.7–89.2%)	*p* < 0.001	0.884	3.734	4.159	13.37	7	9.803	9.493	1.898	—
	MV	3	74.5% (15.1–92.3%)	*p* = 0.020	0.498	—	5.231	11.431	2	—	1.588	2.362	—
van Paassen et al. (2020) (Netherlands)	Mortality	22	60.6% (39.0–74.5%)	*p* < 0.001	0.29	—	4.558	11.546	7	—	20.966	4.422	—
	MV	7	10.9% (0–74.0%)	*p* = 0.346	0.591	—	2.658	2.892	1	—	6.709	6.495	—
Ye et al. (2020) (multiple nations)	Mortality	2	0	*p* = 0.768	—	—	19.911	19.911	2	—	0.6266	0.6266	0.099

**Abbreviations:** AEs, adverse events; 95% CI, 95% confidence interval; ICU, intensive care unit; MV, mechanical ventilation; NA, not available; SD, standard difference; VCT, virus clearance time.

* I^2^ of the heterogeneity test, *p* value for the Q test.

^†^ From Egger’s regression asymmetry test.

^‡^ Observed and expected number of significant studies using effects from the largest study (most with the smallest SE), fixed-effects results, random-effects results of each included meta-analysis as plausible effect size.

^§^ Two-sided *p* value of the excess significance test on intended outcomes. Hospital stay in the study of Lu et al. and mortality in the study of Ye et al. had O > E results, and the *p* value was calculated.

### Small-Study Effects

With the exception of Cano et al. (Cano et al., 2020), who analyzed corticosteroid use on mortality, there was no evidence for the exhibition of small-study effects according to Egger’s test (Table 3). However, there were only five comparisons including no less than 10 studies, which provided enough statistical power to identify the small-study effects through Egger’s test.

### Excessive Significance Bias

O and E for the largest study, summary effect size on fixed effects, and summary effect size on random effects were rigorously calculated. Only O > all three kinds of plausible E with *p* < 0.10 was thought to have excessive significance bias. O > E was found by Lu et al. (Lu et al., 2020) for hospital stay and Ye et al. (Ye et al., 2020) for mortality, *p* = 0.112 and *p* = 0.099, respectively. Therefore, only mortality reported by Ye et al. (Ye et al., 2020) showed evidence of excessive significance bias, although the effect was slight (Table 3).

### Pooled Results From Each Eligible Study

We extracted subgroup outcomes from the eligible studies, and there were critically ill/severe and non-severe patients. Eight meta-analyses (Budhathoki et al., 2020; Cano et al., 2020; Cheng et al., 2020; Pasin et al., 2020; Siemieniuk et al., 2020; Sterne et al., 2020; Tlayjeh et al., 2020; Ye et al., 2020) reported 22 subgroup analyses, and only four comparisons had significant results on the primary effect size, effect size on fixed models, and effect size on random models. Four comparisons of mortality were significant at *p* < 0.05 in the random-effects model: one suggested no benefits from corticosteroids for severe COVID-19 and the other three showed a controversial status regarding the MV-required and no MV/oxygen–required patients (Table 4).

**TABLE 4:** Description of available subgroup results on outcomes of interest reported by included meta-analysis.

Publication	Outcomes	No. of studies	Subgroup	Primary estimate size (95% CI)	Fixed (95% CI)	Random (95% CI)	I^2^ (95% CI)	*p* heterogeneity	95% PI	Egger’s *p* value
Budhathoki et al. (2020) (Nepal)	MV	6	Invasive MV	OR: 1.40 (0.28–6.98)	OR: 1.59 (1.13–2.24)^‡^	OR: 1.40 (0.28–6.98)	91.5% (84.2–95.4%)	*p* < 0.001	0–424.62	0.576
		1	Non-invasive MV	OR: 1.79 (0.35–9.02)	—	—	—	—	—	—
Cano et al. (2020) (America)	Mortality	8	On severe COVID-19	OR: 0.65 (0.51–0.83)	OR: 0.65 (0.51–0.83)	OR: 0.76 (0.48–1.19)	29.3% (0–68.4%)	*p* = 0.195	0.28–2.03	0.205
		2	On high-dose corticosteroid use	OR: 0.57 (0.27–1.23)	OR: 0.57 (0.27–1.23)	OR: 0.57 (0.27–1.23)	0	*p* = 0.939	—	—
		11	On low-dose corticosteroid use	OR: 1.13 (0.71–1.80)	OR: 0.88 (0.78–0.98)	OR: 1.13 (0.71–1.80)	59.7% (21.7–79.2%)	*p* = 0.006	0.32–3.96	0.214
Cheng et al. (2020) (China)	Mortality	4	On severe COVID-19	RR: 1.80 (0.51–6.33)	RR: 2.87 (2.31–3.57)	RR: 1.44 (0.33–6.32)	97.1% (94.9–98.4%)	*p* < 0.001	—	0.749
		2	On non-severe COVID-19	RR: 1.27 (0.43–3.78)	RR: 1.27 (0.43–3.78)	RR: 1.08 (0.27–4.43)	93.6% (79.2–98.0%)	*p* < 0.001	—	—
	VCT	2	On severe COVID-19	WMD: 0.85 (-1.38–3.08)	WMD: 0.75 (-0.60–2.10)	WMD: 0.75 (-0.60–2.10)	0	*p* = 0.322	—	—
		3	On non-severe COVID-19	WMD: 1.43 (-2.19–5.05)	WMD: 2.60 (1.78–3.43)	WMD: 2.97 (-1.65–7.59)	95.0% (71.5–100%)	*p* < 0.001	-115.46	0.834
Pasin et al. (2020) (multiple nations)	Mortality	3	On MV-required patients^†^	RR: 0.85 (0.72–1.00)	RR: 0.80 (0.71–0.91)	RR: 0.85 (0.71–1.02)	66.0% (0–90.0%)	*p* = 0.052	0.11–6.52	0.615
		2	On no MV–required patients*	RR: 0.95 (0.86–1.06)	RR: 0.95 (0.86–1.06)	RR: 0.95 (0.86–1.06)	0	*p* = 0.849	—	—
		2	On no oxygen–required patients*	RR: 1.28 (1.00–1.62)	RR: 1.28 (1.01–1.63)	RR: 1.28 (1.01–1.63)	0	*p* = 0.461	—	—
Siemieniuk et al. (2020) (multiple nations)	Mortality	1	On MV-required patients^†^	OR: 0.59 (0.44–0.78)	OR: 0.59 (0.44–0.78)	OR: 0.59 (0.44–0.78)	—	—	—	—
		2	On no MV–required patients*	OR: 1.05 (0.68–1.60)	OR: 0.94 (0.82–1.08)	OR: 1.05 (0.68–1.60)	85.3% (40.4–96.4%)	*p* = 0.009	—	—
Sterne et al. (2020) (multiple nations)	Mortality	7	On MV-required patients^†^	OR: 0.69 (0.55–0.86)	OR: 0.70 (0.56–0.87)	OR: 0.82 (0.54–1.25)	44.2% (0–76.5%)	*p* = 0.097	0.29–2.30	0.032
		4	On no MV–required patients*	OR: 0.41 (0.19–0.88)	OR: 0.40 (0.19–0.87)	OR: 0.41 (0.19–0.88)	0 (0–43.5%)	*p* = 0.846	0.07–2.20	0.41
Tlayjeh et al. (2020) (Arabia)	Mortality	3	On critically ill COVID-19	RR: 0.80 (0.26–2.46)	HR: 0.68 (0.54–0.85)	HR: 0.80 (0.26–2.46)	84.4% (53.6–94.8%)	*p* = 0.002	0-NA	0.83
		8	On severe COVID-19	RR: 0.98 (0.73–1.30)	HR: 0.89 (0.81–0.98)	HR: 0.97 (0.73–1.30)	82.1% (66.0–90.6%)	*p* < 0.001	0.41–2.34	0.66
		2	On non-severe COVID-19	RR: 0.67 (0.19–2.34)	HR: 1.06 (0.82–1.37)	HR: 0.67 (0.19–2.34)	87.20%	*p* = 0.005	—	—
		9	On high-dose corticosteroid use	RR: 0.95 (0.74–1.22)	HR: 0.90 (0.83–0.98)	HR: 0.95 (0.74–1.21)	78.6% (59.7–88.6%)	*p* < 0.001	0.45–1.99	0.724
		2	On low-dose corticosteroid use	RR: 0.97 (0.07–13.31)	HR: 1.89 (1.05–3.41)	HR: 0.97 (0.07–13.31)	92.9% (76.3–97.9%)	*p* < 0.001	—	—
Ye et al. (2020) (multiple nations)	Mortality	2	On severe COVID-19	HR: 2.30 (1.00–5.29)	HR: 2.30 (1.00–5.29)	HR: 2.30 (1.00–5.29)	0	*p* = 0.768	—	—

**Abbreviations:** 95% CI, 95% confidence interval; COVID-19, coronavirus disease 2019; HR, hazard ratio; MV, mechanical ventilation; OR, odds ratio; 95% PI, 95% prediction interval; RR, relative risk; VCT, virus clearance time; WMD, weighted mean difference.

* No MV–required and no oxygen–required patients were regarded to have non-severe COVID-19.

^†^ MV-required patients were regarded to have critically ill/severe COVID-19.

^‡^ Statistically significant results are marked in bold.

**FIGURE 2:**
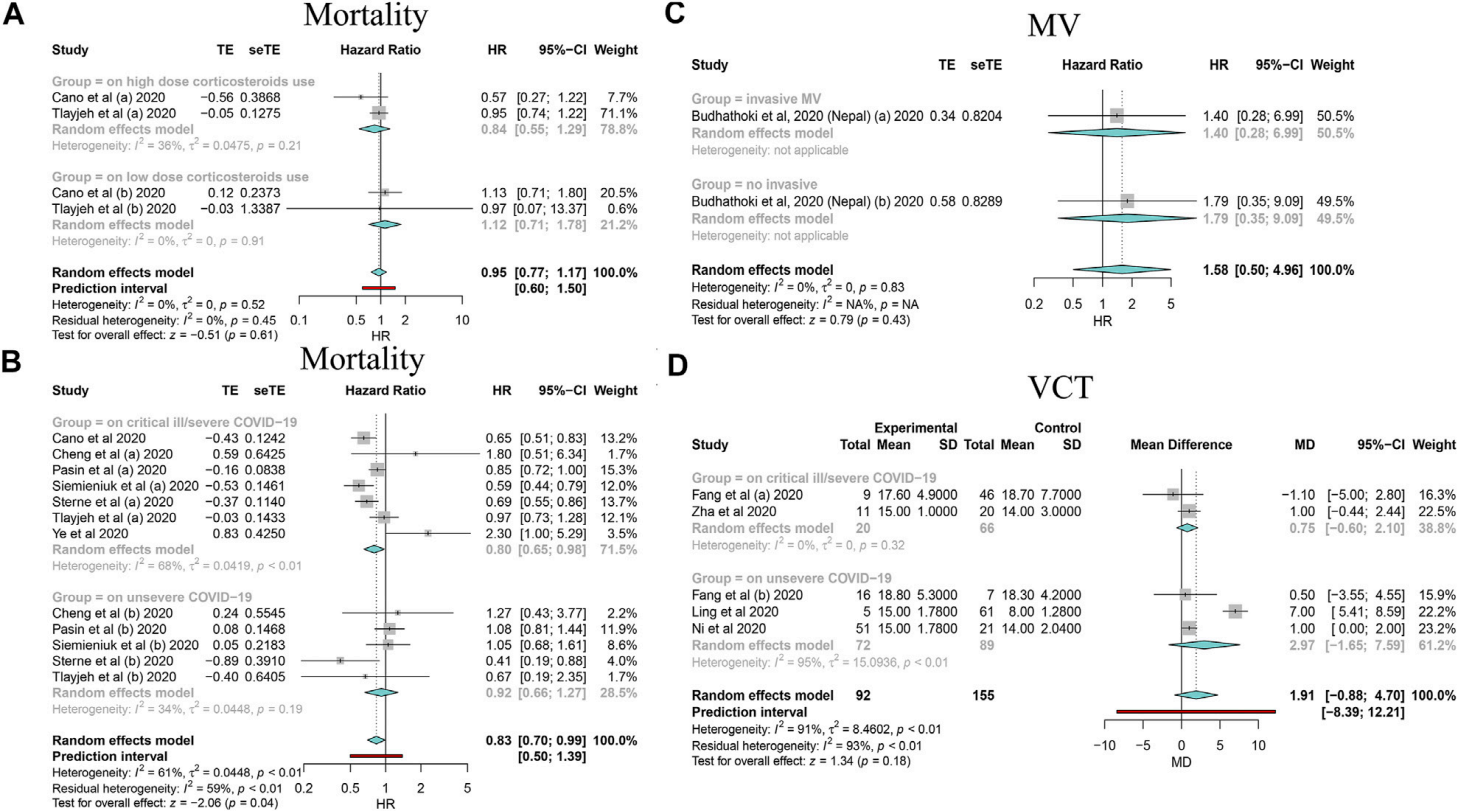
Forest plots based on random effects showing subgroup analysis results on the association between corticosteroid use and mortality, MV, and VCT. **(A)** Subgroup relating to corticosteroid use dose; **(B)** subgroup relating to COVID-19 severity; **(C)** subgroup relating to MV type; **(D)** subgroup relating to COVID-19 severity.

**FIGURE 3:**
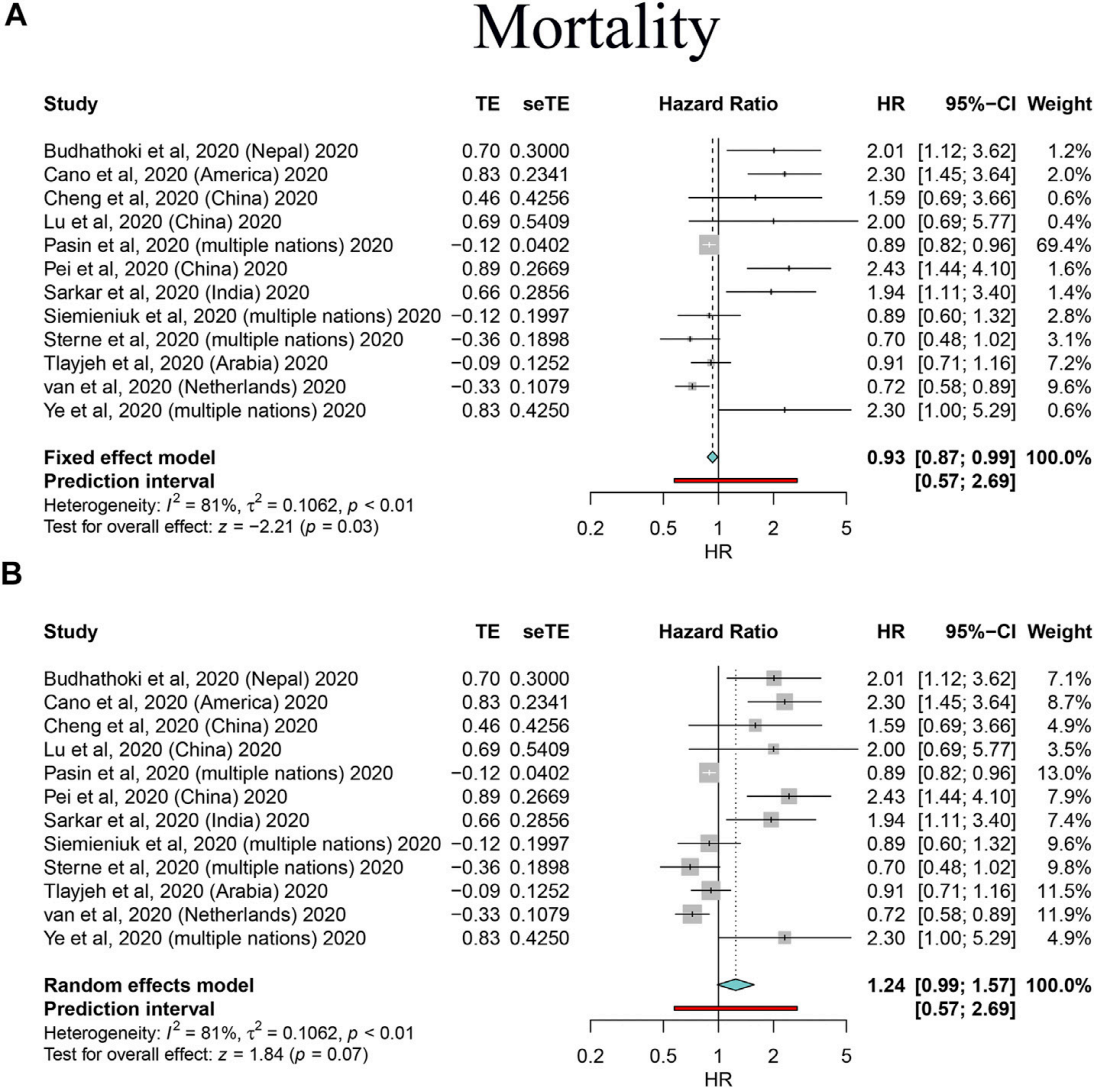
Forest plots based on both fixed and random effects showing main results on the association between corticosteroid use and mortality. **(A)** Results based on the fixed-effects model; **(B)** results based on the random-effects model.

**FIGURE 4:**
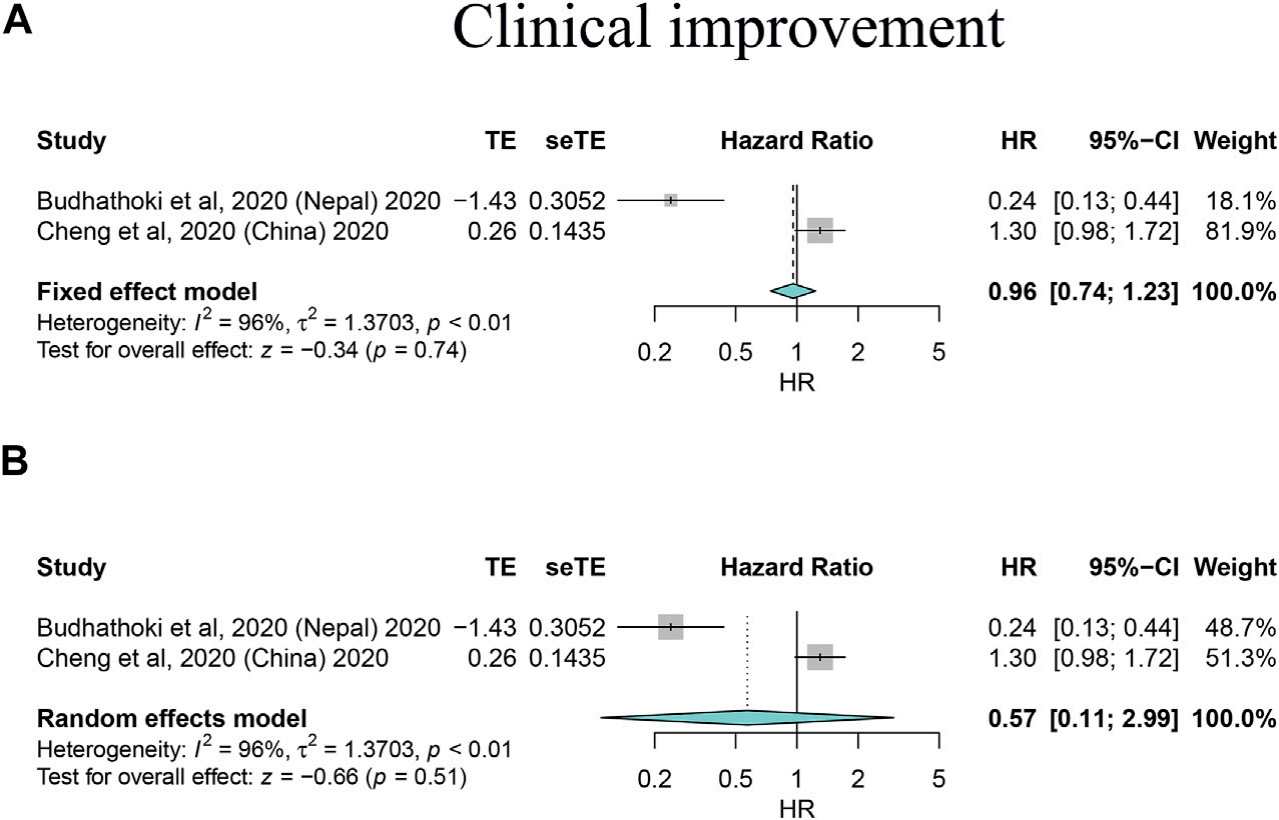
Forest plots based on both fixed and random effects showing main results on the association between corticosteroid use and clinical improvement. **(A)** Results based on the fixed-effects model; **(B)** results based on the random-effects model.

By synthesizing all outcomes of interest together, corticosteroid use was associated with a 27% risk reduction of MV (HR: 0.73, 95% CI: 0.64–0.83; *p* < 0.01 for random-effects models) with low evidence of heterogeneity and with a 20% risk reduction of mortality of critically ill/severe patients (HR: 0.80, 95% CI: 0.65–0.98 for random-effects models) with substantial heterogeneity. Interestingly, we also observed a shortened ICU stay; however, there was a signal of prolonged negative conversion, VCT, and hospital stay in COVID-19 patients who used corticosteroids (Figures 2–4, Table 5, and Supplementary Appendix Figures A1–A3). There was no significant impact of different corticosteroid doses on mortality (HR: 0.84, 95% CI: 0.55–1.29 for high dose; HR: 1.12, 95% CI: 0.71–1.17 for low dose), and a significant impact on other outcomes of interest was not detected.

**TABLE 5:** Summary of pooled outcomes and subgroup analysis results addressed in the study.

Outcomes	Number of included studies	Fixed (95%CI)	Random (95%CI)	Heterogeneity	PI*
Mortality	12^6-17^	HR: 0.93 (0.87–0.99); *p* = 0.03	HR: 1.24 (0.99–1.57); *p* = 0.07	I^2^=81%; *p* < 0.01	0.57–2.69
Clinical improvement	2^6,8^	HR: 0.96 (0.74–1.23); *p* = 0.74	HR: 0.57 (0.11–2.99); *p* = 0.51	I^2^=96%; *p* < 0.01	—
Hospitalization	16	HR: 1.28 (0.27–6.17)	HR: 1.28 (0.27–6.17)	—	—
MV	6^6,8,9,12,13^	HR: 0.73 (0.64–0.83); *p* < 0.01	HR: 0.73 (0.64–0.83); *p* < 0.01	I^2^=0%; *p* = 0.79	0.61–0.87
AEs	1^14^	HR: 0.84 (0.54–1.20)	HR: 0.84 (0.54–1.20)	—	—
Negative conversion	1^6^	WMD: 2.42 (1.31–3.53)	WMD: 2.42 (1.31–3.53)	—	—
VCT	2^8,12^	WMD: 2.02 (1.35–2.68); *p* < 0.01	WMD: 1.87 (-0.27–4.01); *p* = 0.09	I^2^=87%; *p* < 0.01	-14.33
Hospital stay^‡^	4^6,8,9,12^	WMD: 0.98 (0.61–1.34); *p* < 0.01	WMD: 1.39 (-0.84–3.63); *p* = 0.22	I^2^=97%; *p* < 0.01	-19.11
ICU stay	1^8^	WMD: -6.50 (-7.63--5.37)	WMD: -6.50 (-7.63--5.37)	—	—
Stratified analysis^†^					
Mortality					
On high dose of corticosteroid use	2^7,15^	HR: 0.90 (0.71–1.15)	HR: 0.84 (0.55–1.29)	I^2^=36%; *p* = 0.21	Total HR: 0.95 (0.77–1.17); PI: 0.60–1.50
On low dose of corticosteroid use	2^7.15^	HR: 1.12 (0.71–1.78)	HR: 1.12 (0.71–1.17)	I^2^=0%; *p* = 0.91	
On critically ill/severe patients	7^7,8,10,13–15,17^	HR: 0.77 (0.70–0.85)	HR: 0.80 (0.65–0.98)	I^2^=68%; *p* < 0.01	Total HR: 0.83 (0.70–0.99); PI: 0.50–1.39
On non-severe patients	7^7,8,10,13–15,17^	HR: 0.98 (0.79–1.22)	HR: 0.92 (0.66–1.27)	I^2^=34%; *p* = 0.01	
MV					
On invasive MV	1^6^	HR: 1.40 (0.28–6.99)	HR: 1.40 (0.28–6.99)	—	Total HR: 1.58 (0.50–4.96)
On non-invasive MV	1^6^	HR: 1.79 (0.35–9.09)	HR: 1.79 (0.35–9.09)	—	
VCT					
On critically ill/severe patients	1^8^	WMD: 0.75 (-0.60–2.10)	WMD: 0.75 (-0.60–2.10)	—	Total WMD: 1.91 (-0.88–4.70); PI: -8.39–12.21
On non-severe patients	1^8^	WMD: 2.60 (1.78–3.43)	WMD: 2.97 (-1.65–7.59)	—	

**Abbreviations:** AEs, adverse events; 95% CI, 95% confidence interval; HR, hazard ratio; ICU, intensive care unit; MV, mechanical ventilation; NA, not available; PI, prediction interval; VCT, virus clearance time; WMD, weighted mean difference.

* The item included the PI and pooled risk estimate in subgroup analyses.

^†^ Subgroup analysis results.

^‡^ Hospital stay was pooled upon four studies, for Siemieniuk et al. did not provide clear patient sample size.

Great heterogeneity was ascertained in several analyses; thus, we performed sensitivity analyses to control inconsistency and check other sources of bias by omitting heterogeneous studies. We found corticosteroid use was associated with increased mortality in total patients not only in the fixed-effects model (HR: 2.13, 95% CI: 1.69–2.69) but also in the random-effects models (HR: 2.13, 95% CI: 1.69–2.69); however, the use was strongly associated with reduced mortality in critically ill/severe COVID-19 patients (HR: 0.76, 95% CI: 0.68–0.84 for fixed-effects models, and HR: 0.74, 95% CI: 0.63–0.88 for random-effects models), and evidence of negative correlation was even consolidated by sensitivity analyses after omitting studies; prolonged hospital stay still existed and was confirmed (WMD: 3.39, 95% CI: 2.77–4.01 for fixed-effects models, and HR: 3.54, 95% CI: 2.34–4.74 for random-effects models). Overall, our main findings were further consolidated by the sensitivity analyses that also helped to control across-study heterogeneity to some extent (Table 6).

**TABLE 6:** Sensitivity analyses on the summarized pooled outcomes and subgroup analysis results by omitting studies of great heterogeneity.

Outcomes	Number of studies	Fixed (95%CI)	Random (95%CI)	Heterogeneity	PI*
Mortality	7^6-9,11,12,17^	HR: 2.13 (1.69–2.69); *p* < 0.01	HR: 2.13 (1.69–2.69); *p* < 0.01	I^2^=0%; *p* = 0.99	1.57–2.90
Hospital stay	2^6,9^	WMD: 3.39 (2.77–4.01); *p* < 0.01	WMD: 3.54 (2.34–4.74); *p* < 0.01	I^2^=70%; *p* < 0.01 (0.005)	-0.25–7.34
Stratified analysis^†^					
Mortality					
On critical ill/severe patients	5^7,10,13–15^	HR: 0.76 (0.68–0.84)	HR: 0.74 (0.63–0.88)	I^2^=60%; *p* = 0.04	Total HR: 0.79 (0.67–0.93); PI: 0.51–1.23
On non-severe patients	7^7,8,10,13–15,17^	HR: 0.98 (0.79–1.22)	HR: 0.92 (0.66–1.27)	I2=34%; *p* = 0.01	

**Abbreviations:** 95% CI, 95% confidence interval; HR, hazard ratio; PI, prediction interval; WMD, weighted mean difference.

* The item included the PI and pooled risk estimate in subgroup analyses.

^†^ Subgroup analysis results.

### Grading the Evidence

In this part, the effect size *p* value for each comparison was mainly focused on the random effects. Comparing corticosteroid to no corticosteroid use, 14 comparisons (41.2%) were supported by weak evidence, 2 (5.9%) were supported by suggestive evidence, and 1 (2.9%) was supported by highly supported evidence (Table 2). The others were not confirmed. Based on the evidence grade, most comparisons were based on weak evidence; therefore, the interpretation should be made with caution.

## Discussion

As available and affordable immunomodulators, corticosteroid use and the interaction with COVID-19 outcomes of interest are of major interest in the administration of these drugs to COVID-19 patients. This timely umbrella meta-analysis mainly found benefits from corticosteroids on a 27% MV risk reduction and a 20% mortality risk reduction of critically ill/severe COVID-19 patients. Surprisingly, corticosteroid use was also significantly associated with a decreased ICU stay time; however, it seemed to lead to a prolonged hospital stay and a longer VCT and negative conversion time. The dose of corticosteroids had little impact on mortality. Corticosteroids were less correlated with other outcomes, including total mortality, clinical improvement, hospitalization, and AEs. Considering the weak evidence, a precise association should be further estimated in large-scale trials.

In asymptomatic or mild COVID-19, an effective immune response could be elicited; however, in severe SARS-CoV-2 infection, the immune response is always not satisfactory and leads to progressive pulmonary damage in the form of acute respiratory distress syndrome (ARDS) or hyperinflammatory status and subsequent multiorgan dysfunction (Azkur et al., 2020; Tay et al., 2020). During this process, a damage-associated molecular pattern is constructed, which further triggers the release of an array of proinflammatory cytokines and chemokines, including IL-6, interferon-γ–induced protein 10, macrophage inflammatory proteins 1α and 1β, and monocyte chemoattractant protein 1. As more monocytes, macrophages, and T cells assemble, aggressive inflammation is promoted (Huang et al., 2020). Therefore, corticosteroids can serve as a key factor to eliminate the inflammatory microenvironment.

Corticosteroids result in lower mortality among critically ill/severe patients, which is consistent with the real situation of patients in clinical practice (Shang et al., 2020). The results of the RECOVERY trial suggest that the risk of death was reduced by 12.1% among patients prescribed a low dose of dexamethasone and receiving invasive MV at randomization (Horby et al., 2020). However, the RECOVERY trial is limited by methodological flaws such as the absence of stratification and incomplete information associated with mortality, which caused heterogeneity at the randomization level (Normand, 2020). In our study, those patients were assigned to critically ill/severe COVID-19 patients. Dramatically, a large number of ongoing clinical trials involving corticosteroids in critically ill patients with COVID-19 quit recruitment after these findings were publicly available because an equipoise for withholding corticosteroids was no longer present. These real-world results from different clinical and geographic settings suggest that, without compelling contraindications, a rigorous corticosteroid regimen can be considered for critically ill/severe patients with COVID-19 as a component of SOC.

However, the findings contrast with outcomes reported for the administration of corticosteroids among patients with influenza, whose mortality and hospital-acquired infections were improved with corticosteroid administration (Lansbury et al., 2020). In the current study, it was impossible to pinpoint whether the distinct populations were critically ill at the time of randomization. Patients might represent a spectrum of illnesses among patients requiring supplementary oxygen by nasal prongs to those with non-invasive MV supported by a form of high-flow oxygen or positive pressure delivered by a mask (Docherty et al., 2020). Different MV or oxygen–requiring types contributed to the heterogeneity among patients. Another problem is that corticosteroid-induced complications could not be evaluated robustly given the limitations of the available data and various definitions/assessment methods across studies. However, the synthesized AEs were likely to be comparable in both patients randomized to corticosteroids and no corticosteroids based on this study (HR: 0.84, 95% CI: 0.54–1.20).

An interesting finding is that corticosteroid use is associated with a decreased ICU stay time as well as a prolonged hospital stay, VCT, and negative conversion. The decreased ICU stay time suggested that corticosteroids seemed to be effective in critically ill/severe patients with COVID-19, which was also addressed in the current study. Agents of corticosteroids are thought to have higher lung tissue-to-plasma ratios and accordingly can be used in lung injury models (Annane et al., 2017). These agents administrated in the ICU to cure ARDS of critically ill patients were associated with a shorter ICU stay (Annane et al., 2017). Early after the outbreak of COVID-19, the WHO recommended against the routine use of systematic corticosteroids for COVID-19 patients due to acknowledged AEs and a prolonged VCT. Reviewing the AEs, they were found to downregulate immunity, which can explain the prolonged hospital stay and negative conversion (Felsenstein et al., 2020). Similar results in a retrospective study suggested corticosteroids were associated with delayed MERS coronavirus RNA clearance (Arabi et al., 2018). Additionally, studies also showed corticosteroid use was associated with longer throat viral RNA detectability and acute respiratory syndrome coronavirus 2 shedding in mild-to-moderate COVID-19 patients, and clinical benefits on these patients were limited (Jamaati et al., 2021; Tang et al., 2021). Immune response and CD8^+^ T cell infiltration were involved in the COVID-19 pathological process, and reduction of CD4^+^ T cells, CD8^+^ T cells, and NK cells was observed on day 7 after corticosteroid use (Du et al., 2020). The dysregulated immune response and suppressive immune cells, therefore, may contribute to retarding virus shedding and a longer VCT and negative conversion. With the exception of the elusive status of corticosteroids on COVID-19, there are considerable benefits for ARDS, and a meta-analysis showed that the mixed use of hydrocortisone and methylprednisolone offered a reduced time to extubation, a shorter hospital stay, and less mortality with an increase in MV-free days and ICU-free days (Hashimoto et al., 2017). The proposed doses for methylprednisolone are 1–2 mg/kg, followed by the same daily dose at an infusion rate of 10 ml/h daily with a gradual taper in this setting (Meduri et al., 2018; Villar et al., 2020). Another study supported that methylprednisolone at 2 mg/kg is superior than dexamethasone at 6 mg/kg with lower MV and reduced hospital stay due to hypothesized higher lung tissue-to-plasma ratios in methylprednisolone (Ranjbar et al., 2021). An individual patient analysis investigating corticosteroid use on early and late ARDS demonstrated improved survival and a decreased duration of MV (Meduri et al., 2016). Based on current and previous evidence, the Society of Critical Medicine/European Society of Intensive Care Medicine guidelines recommend the early prescription of corticosteroids for moderate-to-severe ARDS (Annane et al., 2017); similarly, the Chinese National Clinical Guidance for COVID-19 Pneumonia Diagnosis and Treatment (available at http://www.nhc.gov.cn/yzygj/s7653p/202003/46c9294a7dfe4cef80dc7f5912eb1989.shtml) published by the Chinese National Health Committee set the initial recommendations for methylprednisolone in patients with clinically progressive deterioration. Other international societies and organizations, such as the American Thoracic Society, the Infectious Disease Society of America, the National Institute of Health of the United States, the Surviving Sepsis Campaign, and the WHO, have also mentioned recommendations for corticosteroids in COVID-19 patients (Cano et al., 2020; Lane and Fauci, 2021).

### Implications and Limitations

To our best knowledge, this is the first umbrella meta-analysis that summarizes the current secondary and quantitative evidence. This study expands the strength of the data and the comprehensive synthesis results, extends the understanding of the role of corticosteroid use, and potentially offers a high-level reference for the management of COVID-19. This study also has several limitations: (1) Most of the incorporated evidence was estimated as weak, and there was considerable heterogeneity among patients involved in each meta-analysis. The performed sensitivity analyses consolidated the robustness of such findings and strongly supported benefits from corticosteroid use on critically ill/severe patients, and we still recommend coming umbrella reviews based on meta-analyses incorporating high-quality RCTs. And the implications of the current evidence need to be combined with the actual clinical situation or validated in high-quality trials. (2) The optimal dose and duration of treatment could not be assessed in this analysis; however, no clear evidence of the effects of different doses of corticosteroids associated with COVID-19 mortality was found in this study. Discrepancies derived from corticosteroid type and administration methods were difficult to address. These problems needed to be further investigated in original studies advent. (3) Some results were limited by missing outcome data, and the definitions of the outcomes of interest were not consistent across the primary studies in each meta-analysis. In the future, the one-dose-fits-all strategy is unlikely to be endorsed, and precise stratification analyses are needed to investigate the proper dose or duration and its real efficacy in pediatrics. It would be interesting to analyze the cost-effectiveness status of wide corticosteroid use on COVID-19 due to its available role in such a pandemic.

## Conclusion

The current umbrella synthesis indicates that corticosteroid use is associated with reduced MV and mortality in critically ill/severe COVID-19 patients, and corticosteroid use leads to a decreased ICU stay but possibly a prolonged hospital stay, a longer VCT, and a longer time to negative conversion. There was no clear evidence of the impact of different corticosteroid doses on mortality. Considering the inconsistency in these results, the benefits and harms of corticosteroids should be reevaluated. Since corticosteroids are affordable and accessible in health care, corticosteroid use may be recommended in critically ill patient care without steadily increasing the total mortality under the pressure of such a rapid global outbreak.

## Data Availability

The original contributions presented in the study are included in the article/Supplementary Material, and further inquiries can be directed to the corresponding author.

## Abbreviations

AEs, adverse events; ARDS, acute respiratory distress syndrome; 95% CI, 95% confidence interval; COVID-19, coronavirus disease 2019; E, expected number of studies; HR, hazard ratio; ICU, intensive care unit; MD, mean difference; MV, mechanical ventilation; O, observed number of studies; OR, odds ratio; RCTs, randomized controlled trials; RR, relative risk; SE, standard error; SOC, standard of care; VCT, virus clearance time; WHO, World Health Organization; WMD, weighted mean difference.
