# Supplementary figures and images for: Systemic Corticosteroid Administration in Coronavirus Disease 2019 Outcomes: An Umbrella Meta-Analysis Incorporating Both Mild and Pulmonary Fibrosis–Manifested Severe Disease
Source: Front Pharmacol. 2021 May 26;12:670170. doi: 10.3389/fphar.2021.670170 (PMC8187793; doi:10.3389/fphar.2021.670170)

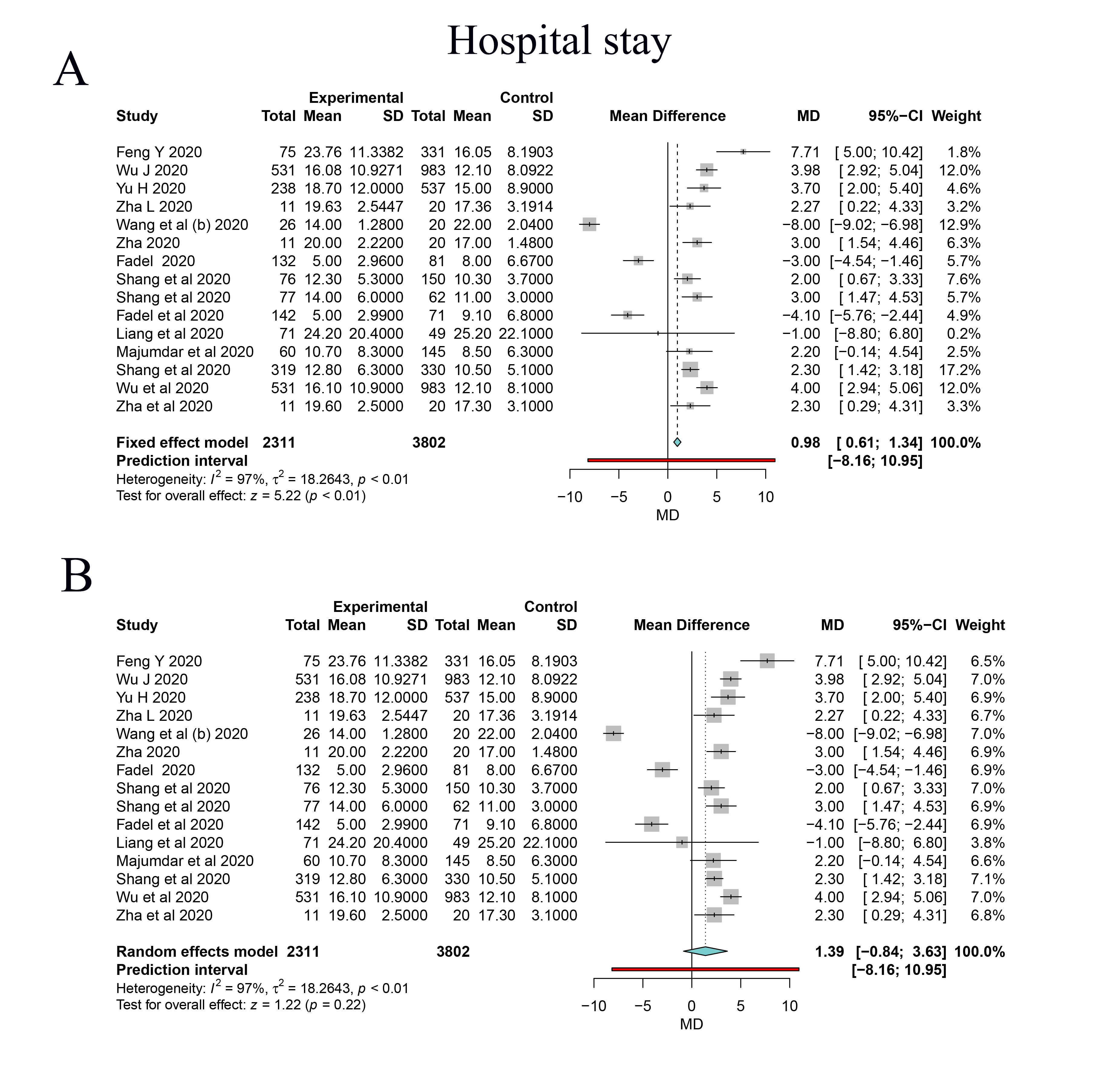

Supplement: Supplementary file 1 [file Image3.TIF]

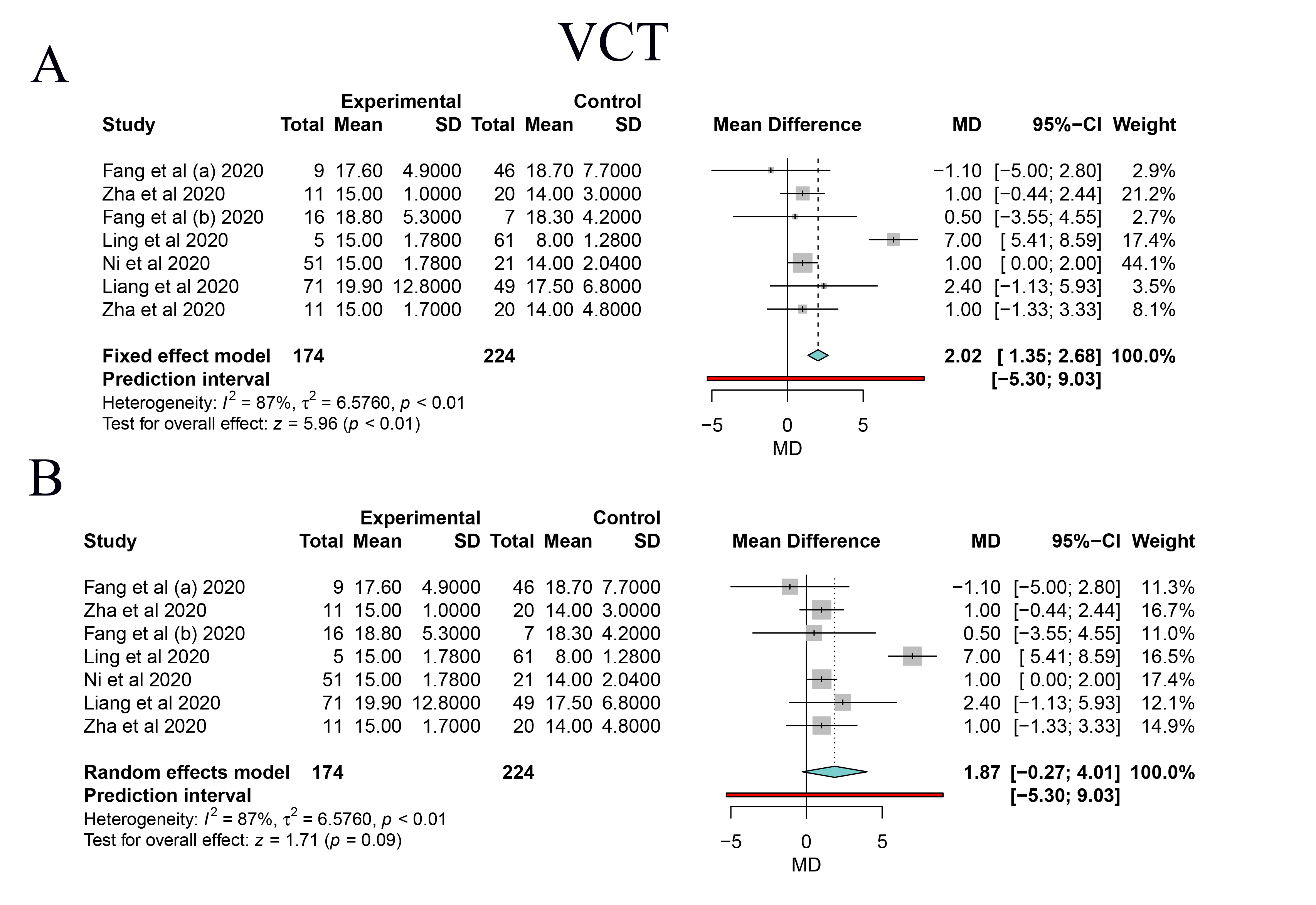

Supplement: Supplementary file 2 [file Image2.TIF]

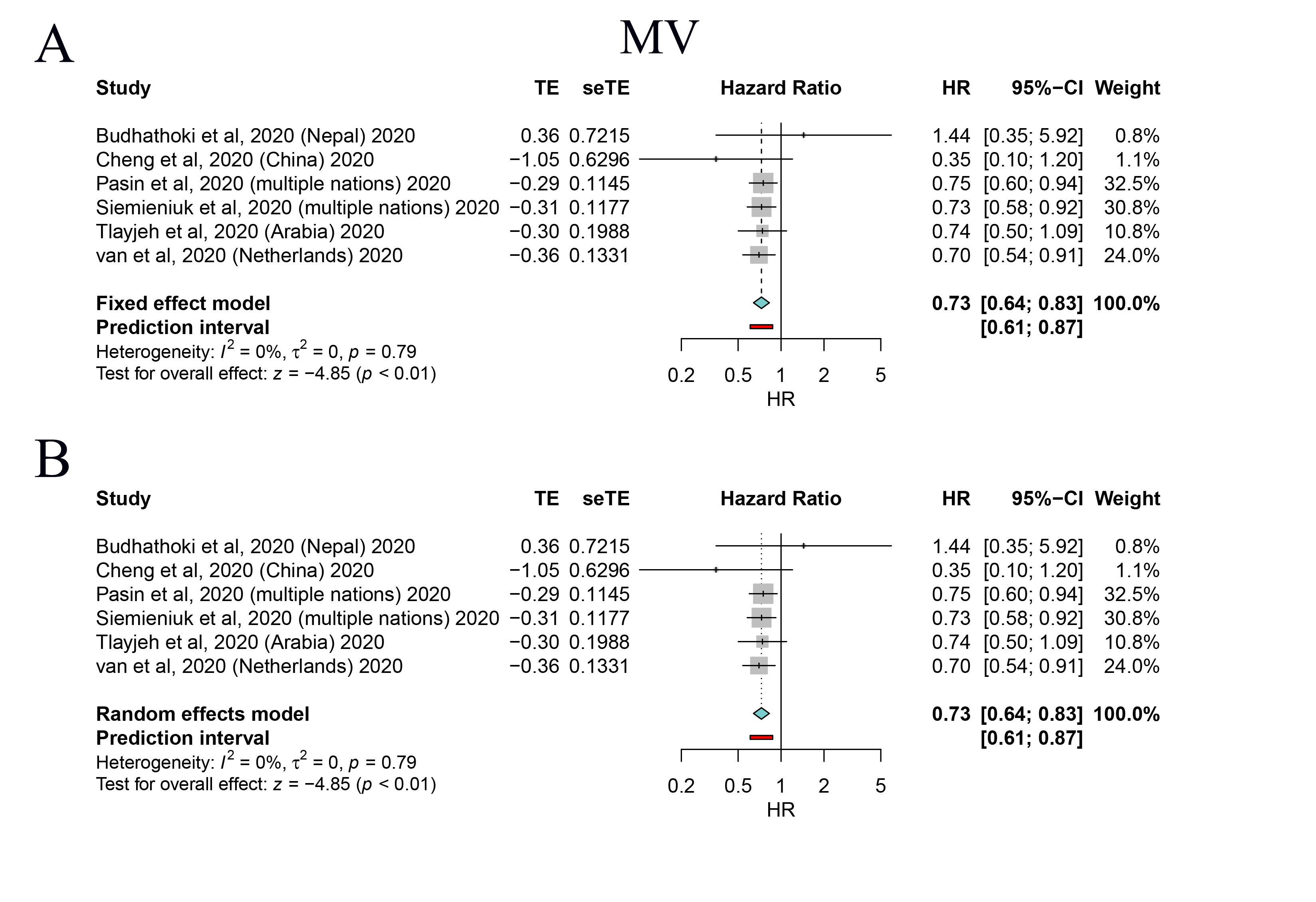

Supplement: Supplementary file 3 [file Image1.TIF]
