# Supplementary material for: Systemic Corticosteroid Administration in Coronavirus Disease 2019 Outcomes: An Umbrella Meta-Analysis Incorporating Both Mild and Pulmonary Fibrosis–Manifested Severe Disease
Source: Front Pharmacol. 2021 May 26;12:670170. doi: 10.3389/fphar.2021.670170 (PMC8187793; doi:10.3389/fphar.2021.670170)
Supplement: Supplementary file 5 [file Table2.DOC]

**Appendix File 3. The complete search strategy**

**The Search Strategy for Eligible Secondary Studies**

Relating published trials were identified after a rigorous literature search on PubMed, EMBASE, Cochrane Library and preprint platforms from inception to Dec-01-2020. The key items used were “COVID-19”, “Adrenal Cortex Hormones”, “systematic review”, “meta-analysis”. No restrictions were applied on language. Reference lists were searched manually for additional records.

# Comprehensive searches were conducted in four electronic databases:

(1) PubMed/Medline (NLM)

(2) EMBASE (Elsevier)

(3) Cochrane Library (CENTRAL/Wiley)

(4) Preprint platforms (medRxiv, bioRxiv)

The literature search strategy was developed first in PubMed and then translated to the other databases. A combination of relevant keywords and controlled vocabulary (MeSH - Medical Subject Headings in PubMed and Emtree in EMBASE) were used in the PubMed and EMBASE searches. Comparable keyword search strategies were used in Cochrane Central Register of Controlled Trials (CENTRAL) and meta-analysis database.

No date or language restrictions were applied. Results were limited to Human studies. MEDLINE records were excluded from EMBASE results sets.

# Four component concepts made up the search strategy:

(1) COVID-19

(2) Adrenal Cortex Hormones

(3) systematic review

(4) meta-analysis

For search set #4, we used Cochrane Handbook recommended search filters for finding systematic review/meta-analysis. Search filters were used for finding systematic review/meta-analysis in PubMed and EMBASE. Available database limiters were used in Cochrane CENTRAL

<http://work.cochrane.org/pubmed>
**sensitivity- and precision-maximizing version (2008 revision); PubMed format**

(systematic review [pt] OR Review, Systematic [pt] OR meta-analysis [tiab] NOT humans [mh])

<http://work.cochrane.org/embase>

**Embase search strategy for finding systematic review/meta-analysis in Embase was the same**

**Preprint platforms search strategy for finding systematic review/meta-analysis in bioRxiv and medRxiv was used with the same key words.**

Each of the four components of the search strategy was first searched upon individually, combining synonyms describing that concept with the Boolean operator OR. The four individual component search sets were then combined together using the Boolean operator AND.

Resulting citations were managed and duplicates removed using the Endnote citation management software program X9 (Thomson Reuters).

**##Search example listed as Pubmed/MEDLINE**

|  | **PubMed/MEDLINE Search Strategy** |
| --- | --- |
| 1. **COVID-19** | ("COVID-19"[Mesh] OR " COVID 19" OR " COVID-19 Virus Disease" OR " COVID 19 Virus Disease" OR "COVID-19 Virus Diseases" OR "Disease, COVID-19 Virus" OR " Virus Disease, COVID-19" OR " COVID-19 Virus Infection" OR " COVID 19 Virus Infection" OR " COVID-19 Virus Infections" OR “Infection, COVID-19 Virus” OR “2019-nCoV Infection” OR “Coronavirus Disease-19” OR “2019 Novel Coronavirus Disease” OR “2019-nCoV Disease” OR “Disease, 2019-nCoV” OR “Infection, SARS-CoV-2” OR “COVID-19 Pandemic” OR “Pandemic, COVID-19”) |
| 1. **Adrenal Cortex Hormones** | ("Adrenal Cortex Hormones"[Mesh] OR " Hormones, Adrenal Cortex" OR " Adrenal Cortex Hormone" OR " Cortex Hormone, Adrenal" OR " Hormone, Adrenal Cortex" OR " Corticosteroids" OR " Corticosteroid" OR "Corticoids" OR “Corticoid”) |
| 1. **systematic review** | ("systematic review" [Mesh] OR "Review, Systematic") |
| 1. **meta-analysis** | (meta-analysis [pt] OR secondary study[tiab] OR secondary studies[ti]) |
| **5** | (animals[mh] NOT humans [mh]) |
| **6** | **1** AND **2** AND **3** AND **4** |
| **7** | **6** NOT **5** |

**Abbreviations:**  **Mesh** = Medical Subject Heading, **pt** = Publication Type, **tiab** = Title/Abstract, **ti** = Title, **mh** = MeSH Terms.
